# Initial Binding of Ions to the Interhelical Loops of Divalent Ion Transporter CorA: Replica Exchange Molecular Dynamics Simulation Study

**DOI:** 10.1371/journal.pone.0043872

**Published:** 2012-08-30

**Authors:** Tong Zhang, Yuguang Mu

**Affiliations:** School of Biological Sciences, Nanyang Technological University, Singapore, Singapore; Russian Academy of Sciences, Institute for Biological Instrumentation, Russian Federation

## Abstract

Crystal structures of *Thermotoga maritima* magnesium transporter CorA, reported in 2006, revealed its homo-pentameric constructions. However, the structure of the highly conserved extracellular interhelical loops remains unsolved, due to its high flexibility. We have explored the configurations of the loops through extensive replica exchange molecular dynamics simulations in explicit solvent model with the presence of either Co(III) Hexamine ions or Mg^2+^ ions. We found that there are multiple binding sites available on the interhelical loops in which the negatively charged residues, E316 and E320, are located notably close to the positively charged ions during the simulations. Our simulations resolved the distinct binding patterns of the two kinds of ions: Co(III) Hexamine ions were found to bind stronger with the loop than Mg^2+^ ions with binding free energy −7.3 kJ/mol lower, which is nicely consistent with the previous data. Our study provides an atomic basis description of the initial binding process of Mg^2+^ ions on the extracellular interhelical loops of CorA and the detailed inhibition mechanism of Co(III) Hexamine ions on CorA ions transportation.

## Introduction

With a concentration as high as 15–25 mM, Mg^2+^ ions take part in a diverse biological functions within living cells [1]. In prokaryotes, Mg^2+^ ion has been linked to the virulence as an essential regulatory signal. In eukaryotes, Mg^2+^ ion has also been shown to influence the DNA and protein synthesis [2], [3]. Three types of transporters, MgtE, MgtA/B and CorA, have been identified to own the ability to mediate transport of Mg ions across bacterial membrane [4]. Among them, CorA has been studied the most. CorA was first identified from *Escherichia coli* genome by Silver and colleagues in 1969 [5] and first cloned from *Salmonella typhimuriom* by Hmiel and colleagues in 1986 [6]. Nevertheless, the crystal structure of CorA remained unsolved until 2006, when three individual groups published the crystal structure of divalent ions bound *Thermotoga maritima* CorA [7], [8], [9]. All the three structures clearly show that the functional form of CorA protein is a funnel-like homopentamer. For each monomer, both N- and C-terminals are located at the cytosol side. The N-terminal cytosolic domain forms a “sandwiched structure” in which 7 beta-sheets locate in between 2 series of alpha-helices (helices 1–3 and helices 4–6). Helix 7, the longest helix in CorA, starts from the cytosolic sandwiched structure, includes the first transmembrane domain (TM1), and ends at the periplasmic side. Helix 8 forms the second transmembrane domain (TM2), and brings the C-terminal end back into cytosol. In pentamer, the channel is surrounded by the five TM1s, and the five TM2s form a ring encircling the channel.

The structural information of the short interhelical loop linking TM1 and TM2 was missing in all the three solved structures, probably due to its high flexibility [7], [8], [9]. The interhelical loop contains the signature motif “GMN” of CorA, and another highly conserved motif “MPEL” in most members of CorA family. Besides, several charged residues are present in the loop [10]. As the loop is exposed to periplasm, it was believed to be essential in initial binding of ions, and possibly substrate selection [7]. Moomaw and Maguire recently applied mutational study on the loop region of *Salmonella enterica* serovar Typhimurium CorA and predicted that the interhelical loops provided initial binding site for hydrated Mg^2+^ ion rather than the dehydrated one [11]. They also proposed that the electrostatic interactions between ions and the negatively charged residues were not essential. On the contrary, Hu *et al*. compared binding of Mg^2+^ ions with a well-known CorA inhibitor Co(III)Hexamine (HexCo) to *Mycobacterium tuberculosis* CorA and concluded that the negatively charged residues in the loop region play important roles in cations recognition [12]. Dalmas *et al*. combined ERP and molecular modelling tools to generate a model for the interhelical loop of *T. maritima* CorA, and found that the negatively charged E316 formed a “negatively charged nest” which fits nicely to a hydrated Mg^2+^ ion [13]. The explicit interactions between the loops and ions, as well as the roles of negatively charged residues in ions binding are, however, still not clear yet.

In this study, we have made efforts to sample the configuration of the interhelical loops of *T. maritima* CorA using extensive replica-exchange molecular dynamics (REMD) simulations. Meanwhile the binding interactions of loops and Mg^2+^ ions as well as HexCo ions have been explicitly explored. With the theoretical methods applied, we try to explain the roles of residues in ions binding. Moreover, by comparing the HexCo and Mg^2+^ ions, we also aim to explain the inhibition mechanism of HexCo on CorA theoretically.

## Model and Methods

### Loop model

**Figure 1:**
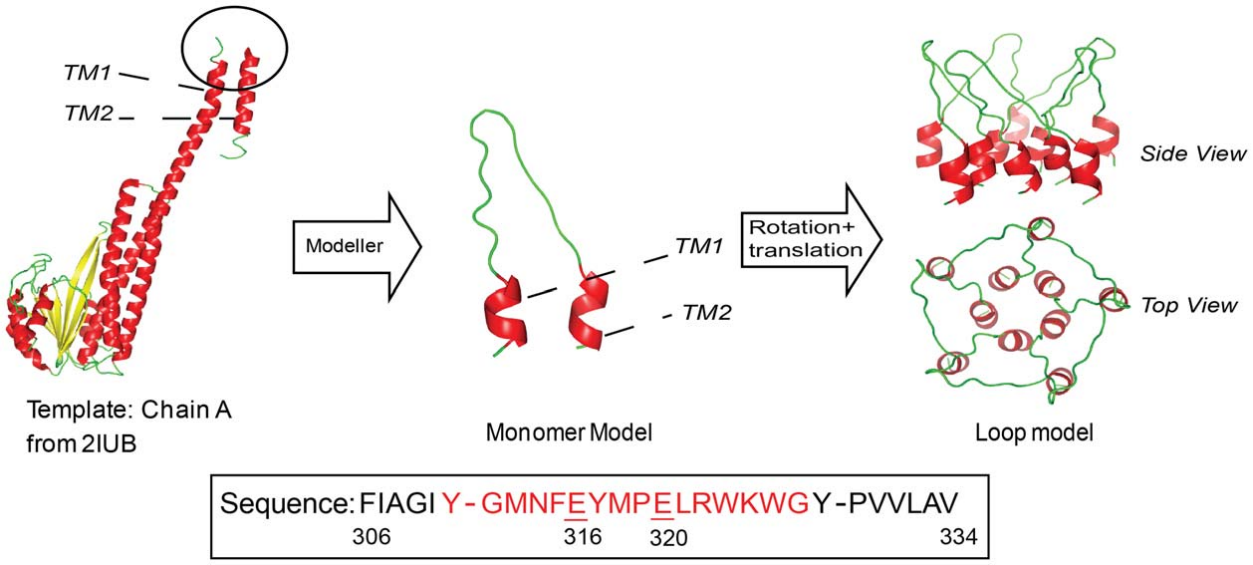
Demonstration of modelling process. The sequence of monomer model, from F306 to V334 is shown. The residues which are missing in 2IUB are shown in red.

The monomer model was built based on homolog modelling method by Modeller software (version: 9v6) [14]. The reference structure used is the Chain A of the crystal structure of CorA solved by Eshaghi et al. (PDB code: 2IUB) with the missing residues from Y311 to G326 (sequence: YGMNFEYMPELRWKWG). Modeller constructed the coordinates of the missing residues according to de novo loop modelling technique. The resultant monomer model with twenty-eight residues, F306 to V333, was denoted as chain A in loop model. Chain A was then rotated and translated symmetrically to get Chain B, C, D and E which together make up the homo-pentameric loop model. The modelling process is shown in Figure 1 schematically. The additional first 6 and last 6 residues, a part of transmembrane helix 1 (TM1) and 2 (TM2), respectively (10), were also included in the model.

### Simulation methods

All molecular dynamics simulations were performed with GROMACS software package version 4.0.3 [15] using the AMBER99SB force field [16]. To enhance sampling, replica exchange molecular dynamics simulations were implemented [17], [18]. Three systems were built up with x-axis parallel to the axis of channel. System 1 is the loop model explicitly solved with 4074 SPC water molecules [19] in a dodecahedron box with an initial volume of 149.746 nm^3^ (abbreviated as LOOP system for short). System 2 is the loop model and 10 HexCo ions explicitly solved with 3992 SPC water molecules in a dodecahedron box with an initial volume of 152.97 nm^3^ (abbreviated as COH system for short). System 3 is the loop model and 10 hydrated Mg^2+^ ions explicitly solvated with 4093 SPC water in a dodecahedron box with an initial volume of 156.66 nm^3^ (abbreviated as MG system for short). 5, 35 and 25 chloride ions were added in LOOP, COH system and MG system respectively to make the systems neutral. The initial topologies of the HexCo were generated in Spartan and energy-minimized [20]. The conformations were further optimized at the HF/6-31G* level using Gaussian09 [21], and the atomic partial charges were derived using R.E.D III package [22]. The parameters for HexCo used in this study, shown in Table S1, is the same with a published work by Cheatham and Kollman in 1997 [23]. To validate the parameters assigned, the RDF of water-HexCo/Mg^2+^ ion and the PMF of the distances between HexCo/Mg^2+^ and glutamic side chain were calculated. The detail of the calculation is provided in Supporting Information. (Section S1 and S2, Figure S1 and S2). For Mg^2+^ ion and chloride ions the CHARMM parameters optimized by Roux and co-workers were used [24]. The combination rule of CHARMM and AMBER force fields for the non-bonded interaction is the same. All 3 systems containing 48 replicas were simulated for 200 ns with the temperature range from 315.0 K to 455.8 K. The average replica exchange ratios are ∼39.1%, ∼37.9% and ∼36.4% for the LOOP, COH and MG systems, respectively. The heavy atoms from residues of TM1 and TM2 were constrained to mimic the effect of membrane at all simulation stages. The rest residues in the loop domain, G312 to Y327, were left to move freely. LINCS protocol [25] was applied to constrain the length of all bonds including hydrogen atoms. The integration step in the simulation is 0.002 ps, and the trajectory was output every 500 integration steps. The replica exchange was attempted every 1000 integration steps, 2ps. Coulomb interactions were treated with the particle mesh Ewald method [26] with the cutoff of 0.9 nm. The van der Waals interactions were treated with the cutoff of 1.2 nm.

### Analysis methods

Trajectories from all simulations were analyzed using GROMACS software package. The commands g_mindist and g_dist were used to calculate minimum distances and inter-centre of mass distances respectively. The command g_gyrate was used to calculate the radius of gyration. The command g_traj was used to output the coordinates of certain groups of atoms. The command g_sas was used to calculate the solvent accessible surface areas (SASA). The command trjorder was used to calculate the number of water molecules around a certain group. Pymol program was used for visualization. Potential of mean force (pmf) was applied to construct the free energy surface in both 1-dimension and 2-dimension. The free energy is given by
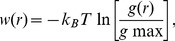
(1)Where, *w(r)* is the Helmholtz free energy, *k_B_* is the Boltzmann constant, T is the temperature in Kelvin, *g(r)* is the sample counts at distance r, and *gmax* is the maximum sample count.

The binding energies were analyzed by the generalized Born model with surface area modification (GBSA) using sander module in the AMBER9 package [27]. The force field used were the same as the one used for the REMD simulations. The GB model developed by Onufriev *et al*, [28] were applied here, and the α, β, and γ were 1.0, 0.8, and 4.85 respectively. Each structure of the REMD simulations produces three copies with all water molecules discarded (the water molecules bound to Mg^2+^ ions were kept): one is the whole system, one with only ions and one with only protein. All of them were inputted into sander module and related energies were output. The energy differences between the system and the sum of separated ions and protein were taken as the binding energy.

## Results

### Convergence check

**Figure 2:**
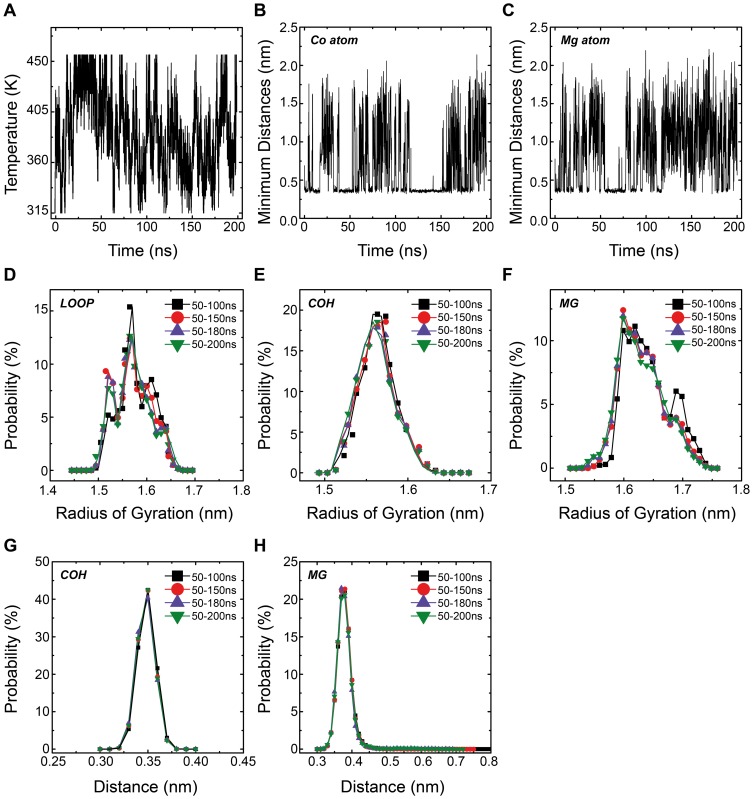
The convergence check. Trace in temperature space of representative replicas (A); Evolution of distances between HexCo ions and protein heavy atoms for representative replicas (B); Evolution of distances between Mg^2+^ ions and protein heavy atoms for representative replicas (C); Distribution of radius of gyration of protein with time interval 50–100 ns (black square), 50–150 ns (red circle), 50–180 ns (violet triangle), and 50–200 ns (green inverse triangle) at 315 K for LOOP (D), COH (E) and MG (F). Distribution of distances between ions and protein heavy atoms with time interval 50–100 ns (black square), 50–150 ns (red circle), 50–180 ns (violet triangle), and 50–200 ns (green inverse triangle) at 315 K for COH (G) and MG (H) system.

REMD simulations enhance the sampling through overcoming potential energy barrier at high temperatures. It is critical to check whether each replica has visited all the temperature ladders several times during the whole trajectory. Figure 2A shows the temperature evolution of one representative replica trajectory over the 200 ns simulation. It is clear that the replica visited a wide range of temperatures. The similar behaviour was also found on other replicas, of which the data are not shown. Another reason to apply REMD method in our simulation is to avoid trapping of bound ions to the protein due to the strong electrostatic interactions. To monitor it, the minimum distances between ions and protein were traced within each single replica. Figures 2B and 2C show the changes of minimum distances between one of the 10 HexCo ions and the protein, and between one Mg^2+^ ion and the protein within the 200 ns trajectories. One observes that the particular Mg^2+^/HexCo ion keeps moving rather than being trapped in binding to the protein. Similar behaviour was also observed on the other ions, of which the results are not shown.

In our systems, the transmembrane domains of the protein were frozen and only the loop domains were allowed to move freely. To depict the flexibility of the loops, a global structural character, the radius of gyration (Rg), of the loop domains was employed. Thus, the convergence of the protein structures was checked through the evolution of distributions of Rg of the protein at the lowest temperature (315 K). Four Rg distribution functions sampled from LOOP, COH and MG systems over different time intervals were plotted in Figure 2D, 2E and 2F, respectively. For the LOOP system, the distribution curves of Rg over different time intervals, 50–100 ns, 50–150 ns, 50–180 ns, 50–200 ns, are plotted in Figure 2D. Among them, the curves for 50–180 ns, 50–200 ns are well overlapped, in terms of the heights and positions of peaks. For the COH system, the distributions of Rg over different time intervals, 50–100 ns, 50–150 ns, 50–180 ns and 50–200 ns, are sharing the similar pattern, among which the curves for 50–180 ns and 50–200 ns are very well overlapped (Figure 2E). For the MG system, the distribution curve for the sampling of 50–100 ns is slightly different from those of 50–150 ns, 50–180 ns and 50–200 ns, with an additional peak at Rg = 1.7 nm. All the three curves sampled over longer time are nearly identical, particularly for the position of peaks (Figure 2F). The evolution of distributions of Rg indicates that the simulations have converged through the 200 ns simulation time in the context of structure of the loops.

One of the major goals in our study is to interrogate the interactions between ions and the protein. Therefore, the convergence was also tested by the distribution of the minimum distance between ions and protein heavy atoms in different time interval from the trajectory at the lowest temperature (315 K). Four distribution curves of the minimum distance between ions and protein heavy atoms of the trajectories at 315 K over different time intervals were plotted in Figures 2G and 2H. For the COH system, the distributions of the minimum distances over 50–100 ns, 50–150 ns, 50–180 ns, and 50–200 ns are showing similar patterns (Figure 2G). Particularly, the curves for 50–150 ns, 50–180 ns and 50–200 ns are nearly identical, which indicates the simulation is well converged within 200 ns. For the MG system, the distributions of the minimum distances within50–100 ns, 50–150 ns, 50–180 ns and 50–200 ns, are plotted in Figure 2H. Same as the COH system, high similarity among the four curves are observed. Again, this shows the MG system is well converged. One phenomenon should be noted that in contrary to the MG system, the curves of 50–150 ns, 50–180 ns and 50–200 ns of the COH system are almost coincident with each other, indicating that COH system converged faster than MG system. Additionally, the long tails appeared in the MG system are lost in the COH system. This indicates that HexCo ions are closer to protein than Mg^2+^ ions. In brief, the simulation systems are well converged within 200 ns which enables further analysis.

### Distribution of the radius of gyration of the loops

Comparing the Rg distribution curves for 50–200 ns of LOOP, COH and MG systems plotted in Figures 2D, 2E, and 2F, one observes that the loop domains in LOOP system have 3 peaks at 1.52 nm, 1.57 nm and 1.64 nm. While in COH system and MG system, there is only one dominant peak in each system. Moreover, the loop domains in the COH system have a narrower distribution with a smaller Rg than that of the MG system. Therefore, the observation indicates that HexCo ions may be more efficient to limit the flexibility of the loops than Mg^2+^ ions due to different binding affinities or binding patterns between HexCo and Mg ions, which will be discussed in later sections.

**Figure 3:**
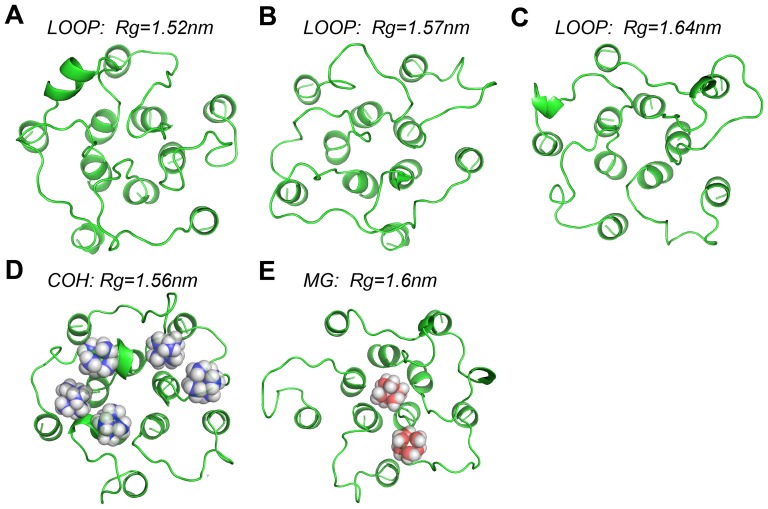
Representative loop/loop-ions structures with distinct Rg values (A, B, C).

In Figures 3A, 3B, and 3C, three snapshots of the loop structure with Rg of 1.52 nm, 1.57 ns, and 1.64 nm respectively are shown. In Figure 3D, a snapshot of the loop structure with Rg of 1.56 nm (the peak position of the Rg distribution) in the COH system is shown. Five tightly bound HexCo ions are found there. In Figure 3E, a snapshot of loop structure with Rg of 1.6 nm is displayed, together with two closely contacted Mg^2+^ ions. With smaller values of Rg, the loops seem be glued together by the ions (see Figure 3D) and with large values of Rg, the loops tend to tilt away from the channel axis (see Figure 3E). Generally speaking, there are very few regular secondary structures, such as α-helices and β-strands, formed in the loops (the results of secondary structure assignment using DSSP method are shown in Table S2).

### Conformational space of the loop structure sampled in different systems

**Figure 4:**
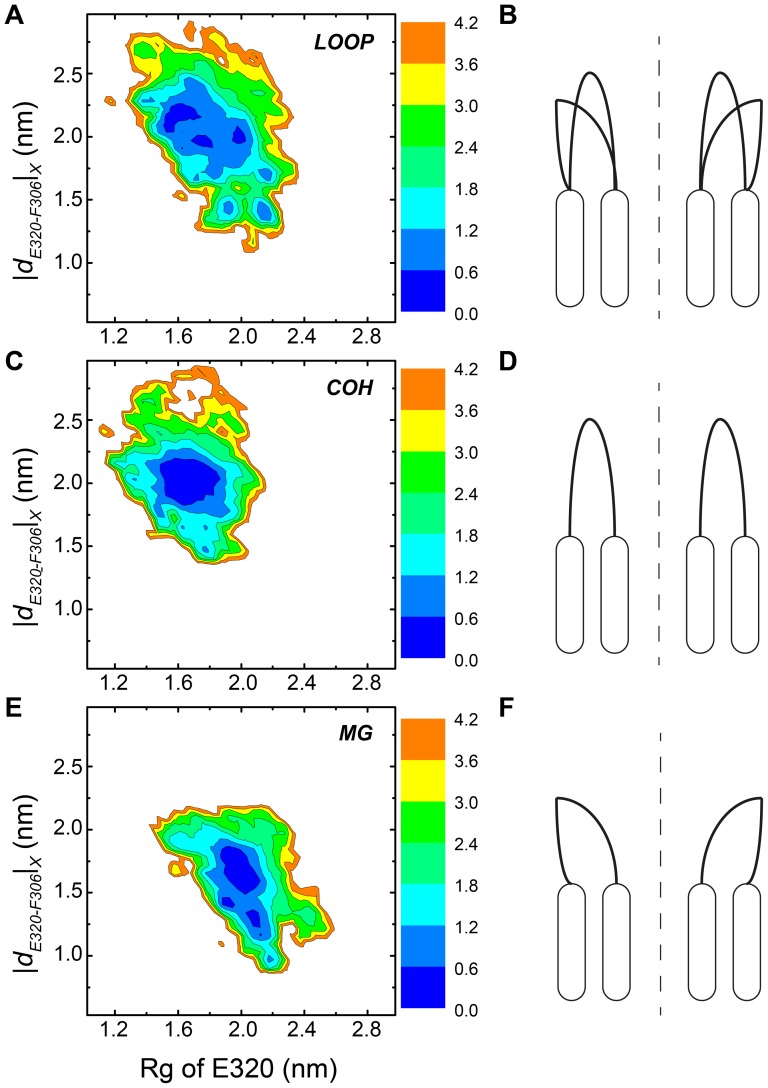
Free energy landscapes at 315 K for LOOP (A), COH (C) and MG (E) systems. The reaction coordinates are Radius of gyration of heavy atoms from E320, and the inter-centre of mass distance between E320 and F306 in X-axis. Sketches of representative structures for LOOP (B), COH (D) and MG (F). Rounded rectangles represent the TM1 and TM2 domains. Black solid lines represent the loop domains. Black dotted lines represent the channel axis.

To further investigate the conformational variability of the loop structures, the free energy surfaces constructed by PMF methods using two reaction coordinates (RC) are shown in Figures 4A (LOOP system), 4C (OCH system) and 4E (MG system). One RC (RC1) is the Rg of heavy atoms from five residues E320. The other RC (RC2) is the inter-centre of mass distances between residues F306 and E320 in X direction. The Rg of residues E320 provides a measure of the opening level of the channel entrance, since residues E320 are located right in the middle of the loop domain. The inter-centre of mass distance between residues F306 and E320 in X direction reflects the height the pentameric loop model from bottom (F306) to top (E320), because the systems were build with x-axis parallel to the axis of channel. One observes the conformational space in LOOP system (Figure 4A) is broader than that of COH (Figure 4C) and MG (Figure 4E) systems, which indicating the loops in LOOP system are more flexible, and HexCo/Mg^2+^ ions function to limit the flexibility of the loops.

The conformations in COH system are mostly populated in the area where RC1 roughly equals to 1.75 nm and RC2 roughly equals to 2.0 nm. While, the conformations in MG system are populated in the area where RC1 roughly equals to 2.0 nm, and RC2 roughly equals to 1.6 nm. The results indicate in MG system, the structures were wider in channel entrance, and shorter in height, comparing with the structures in COH system (Figures 4D and 4F). In LOOP system, both types of structures were present, with different populations though (Figure 4B).

### Distance between ions and the amine groups on loops

**Figure 5:**
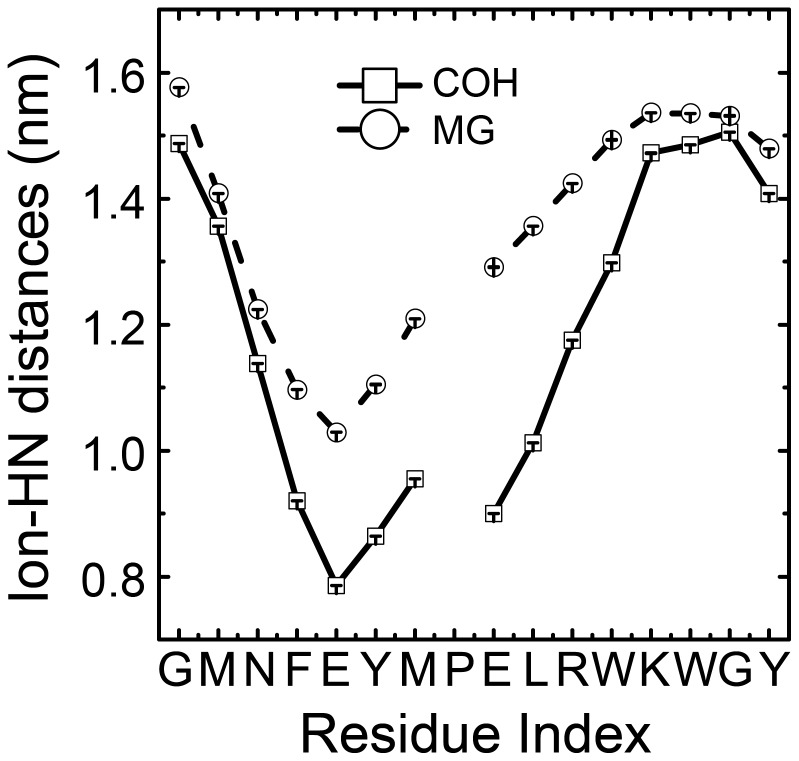
The averaged minimum distances from hydrogen atoms form amine group of interhelical loop residues and the HexCo (black solid line) and Mg^2+^ (black dotted line) ions.

In a NMR study by Hu in 2009 [12], the inter-helical loops of *Mycobacterium tuberculosis* CorA with the presence or absence of Co^2+^ was studied using H/D exchange method. The results revealed the chemical shifts of amide hydrogen atoms of some residues are more sensitive to ions probably due to the nearby ions perturbing the local chemical environment. To check whether present simulations fit their results, the minimum distances between the Mg^2+^ and Co atoms in HexCo to the Hydrogen atom of amine group from residues of the loops were calculated and plotted in Figure 5. In our calculations, Mg^2+^ and HexCo showed similar patterns. From Figure 5, the residues from F315 to L321 have shorter distances than other residues in the interhelical loop, in both COH and MG systems. The results from Hu et al indicated that the Mg2+, Co2+ and HexCo have same binding sites on CorA loops and the most sensitive residues to Co2+ ions are F332 and M333, which corresponding to Y317 and M318 in *Thermotoga maritima* CorA used in the present study. Besides F332 and M333, other residues, from F330 to D337 (F315 to R322 in *Thermotoga maritima* CorA), are also sensitive to Co2+. The R322 here is not near to the cations because of its positive charge, while in the experiment the corresponding residue D337 is close to Co^2+^ since it is negatively charged. The residue E316 and corresponding residue H331 in the experiment also show different behaviours, probably resulting from different charge states. Except R322 and E316, the distance profile of other residues from F315 to L321 agrees well with the experimental results of Hu *et al*.

### Direction of E315 side chains

**Figure 6:**
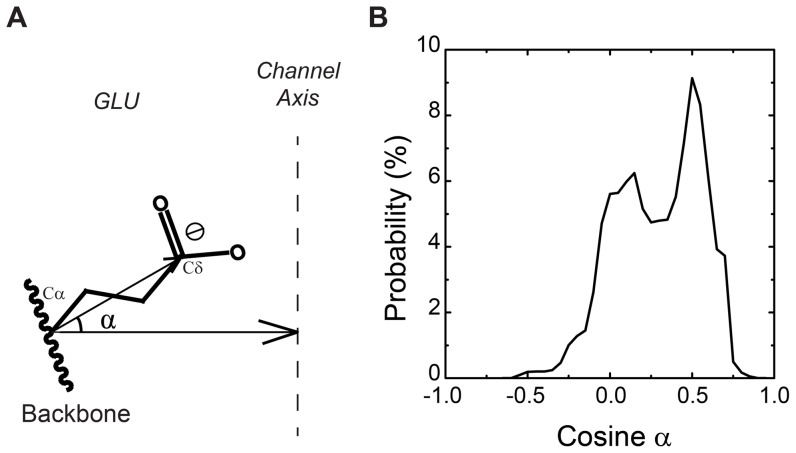
Direction of E316 side chains. Schematic drawing of the vectors from Cα to Cδ within GLU residue and from Cα of GLU to channel axis (A). The distribution of the value of cosine of the angle between the two vectors (B).

In their study Dalmas *et al*. developed an extracellular loop model of CorA by combining electron paramagnetic resonance (EPR) and theoretical modelling method [13]. One of the results was that the residues E316 was located towards the centre of the pentameric ring and together they form a “negatively charged nest” for hydrated Mg^2+^ ions to bind. To check if our model fits Dalmas' results, we defined 2 vectors: One is the vector starting from the alpha carbon ending at the delta carbon of one residue E316; the other one is starting from the alpha carbon of the same E316 pointing to channel axis and is perpendicular to the channel axis. A schematic drawing of the 2 vectors is shown in Figure 6A. The angle α between the 2 vectors was used to define the orientation of the side chain of E316. If the cosine value of the angle α is larger than 0, the angle of the 2 vectors is an acute angle, indicating the side chain of E316 is pointing towards the channel axis. The distribution of the cosine values is shown in Figure 6B. The frames used in the plot are those in which there are Mg^2+^ ions located less than 0.5 nm away from protein surface. The distribution shows a bimodal feature: the higher peak sits around 0.50 relating to an angle of 60 degree between the two vectors; the other peak is around the value of 0.15 corresponding to a side chain configuration with 81 degree of the side chain with respective to the channel axis. Clearly in the simulations the side chains of E316 are very flexible. The distribution highly populated around 0.5 indicates that the side chain of E316 is tilted to the axis of the channel instead of pointing away from the symmetry axis which is consistent with Dalmas' postulation.

**Figure 7:**
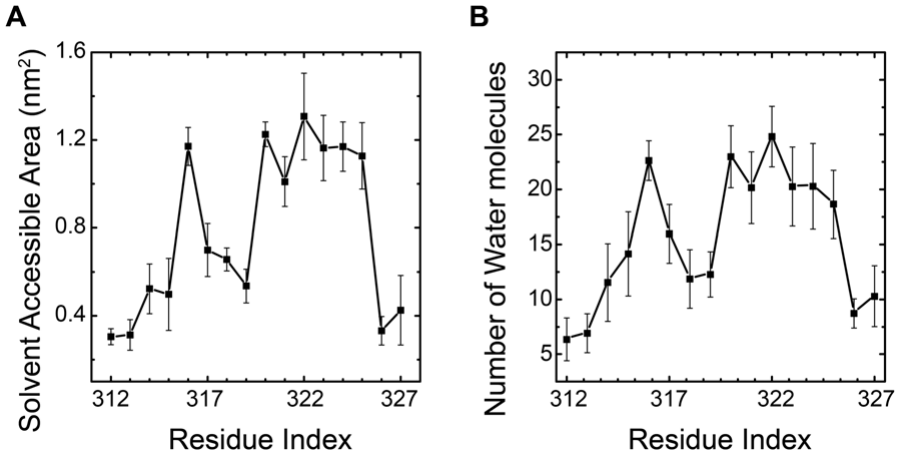
Solvent accessibility. Averaged solvent accessible surface area for residues from G312 to Y327 (A). Ensemble averaged number of water molecules located within 0.5 nm shell from every residue in between G213 to Y327 (B). Both of the two sets of data were calculated from ensemble trajectory at 315 K.

Dalmas *et al*. have also evaluated the solvent accessibility of CorA using fast relaxing agents, NiEdda and O_2_, soluble in water and lipid respectively. Their NiEdda accessibility curve shows two regions where high NiEdda accessibility is observed. The first region is from E316 to Y317, and the second is from L321 to W325, with an exception of a low value at R322. To compare with their results, we calculated the solvent accessible surface area (SASA) and the average number of nearby water molecules (NW) for each residue from G312 to Y327, and the results are shown in Figure 7A and 7B respectively. From the SASA results in Figure 7A one observes two distinct regions with high solvent accessible surface area: first is from F315 to Y317, and second is from E320 to W325. A similar trend can be found in the statistics of NW in Figure 7B. Our results are reasonable in line with Dalmas' experiment, considering the regions with high accessibility. However, the 3 charged residues, E316, E320 and R322, have higher values of SASA and NW value, but low NiEdda accessibility found in the experiment. This is possibly because they used the cysteine scanning technique, in which each residue was replaced by a cysteine and an underestimation of electrostatic interactions between the charged residues and water molecules may happen. Another reason could be that the large size of NiEdda may cause underestimation of the actual water accessibility near sterically hindered region. Generally speaking, our SASA and NW results reflect the trends of the accessibility profiles for the loop and are consistent with experiment done by Dalmas.

### Local binding pattern of ions

**Figure 8:**
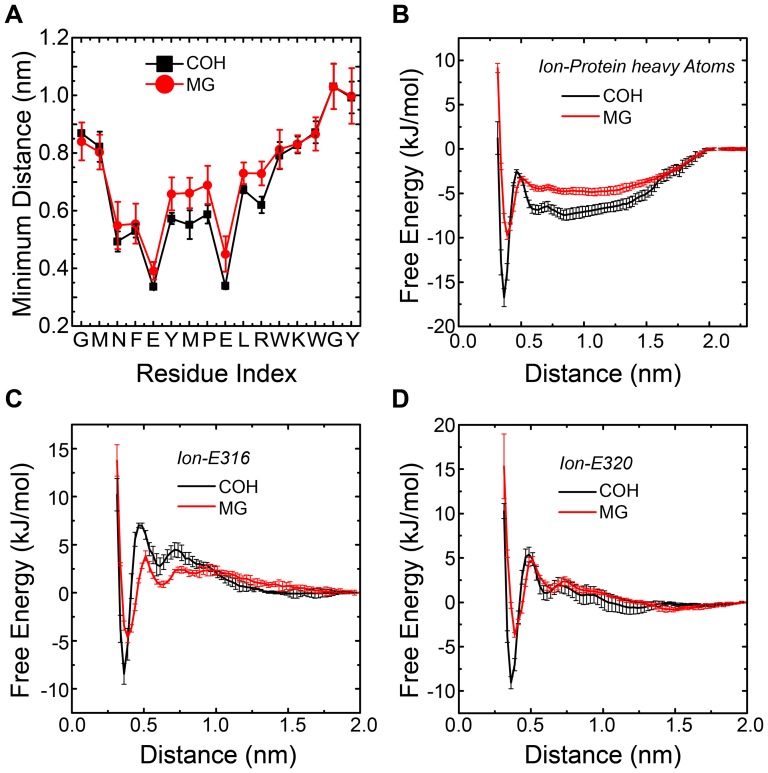
Local binding pattern of the ions. Averaged distances between ions and heavy atoms from residues of loop domain (M313 to Y327) for COH system (black square) and MG system (red circle) calculated from ensemble trajectory at 315 K (A). Potential of mean force as function of distance from ions to protein heavy atoms (B), to E316 heavy atoms (C) and to E320 heavy atoms (D) for COH system (black line) and MG system (red line).

In order to gain insight into the binding pattern of ions on the loops, the averaged minimum distance from Co or Mg atoms to each residue at 315 K was calculated and plotted in Figure 8A. It is natural to think that the residues which were involved in binding with ions would have short distances to the ions. As shown in this figure, HexCo ions have an averaged minimum distance of 0.336 nm to E316 and 0.339 nm to E320, while for Mg^2+^ ions the distances are 0.392 nm and 0.490 nm, respectively. The lowest average minimum distances for MG system, 0.392 nm and 0.490 nm here, are larger than the radius of equivalent hydrated sphere of Mg^2+^ ions which is 0.3 nm [29], indicating that in our system, the Mg^2+^ ions bind to protein in the form of hydrated ions, which correlates well with Moomaw and Maguire's work in 2010 [11]. Additionally, the results also show E316 and E320 play important roles in ion binding. To further study the role of the two glutamic acids, the potential mean forces (PMF) as a function of distances from the Mg or Co atoms to the heavy atoms of the whole protein, of only E316 and of only E320 were calculated based on the equation 1, and the results were plotted in Figure 8B, 8C and 8D respectively. The free energies calculated were then normalized at distance equals 2 nm, where the interactions in between could be ignored. For the COH system, the global minimum is located at 0.37 nm. The binding free energy of HexCo ions to the whole protein, only E316 and only E320 is estimated to be −16.8 kJ/mol, −8.2 kJ/mol and −9.4 kJ/mol, respectively. Clearly the binding of HexCo ions to the loop is dominantly contributed from the two residues, E316 and E320. For MG system, the global minimum is located at 0.39 nm. The binding free energy of Mg^2+^ ions to the whole protein, only E316 and only E320 is estimated to be −9.5 kJ/mol, −4.7 kJ/mol and −4.4 kJ/mol, respectively. The binding of HexCo to the loops is stronger than Mg^2+^ ions, with the binding free energy −7.3 kJ/mol lower.

The ability of HexCo ions to inhibit transportation of Mg^2+^ ions by CorA has been studied by Kucharski *et al*. [30]. They found that the IC_50_ values for Mg ions in the presence of 200 µM Ni^2+^ ions are 1 µM for archeon *M. jannaschii* CorA, and 0.5 µM for *S. typhimuriom* CorA, while the values of HexCo are 200 µM for *M.jannaschii* CorA and 10 µM for *S. typhimuriom* CorA. Based on the Cheng-Prusoff equation [31], 
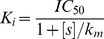
, where *K_i_* is the binding affinity of the inhibitor, [s] is the substrate concentration, *K_m_* is the concentration of substrate at which enzyme activity is at half maximal, and 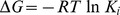, where ΔG is the binding free energy, R is the gas constant and T is the temperature, we can get the binding free energy difference, ΔΔG, between HexCo-CorA and Mg^2+^-CorA, is around −5.9 to −7.7 kJ/mol at T = 310 K (the experimental temperature). Suppose that the binding sites of HexCo and Mg^2+^ ions are both on the extracellular loops, our simulation PMF data, ΔΔG = −7.3 kJ/mol, is consistent very well with the experimental measurements.

**Figure 9:**
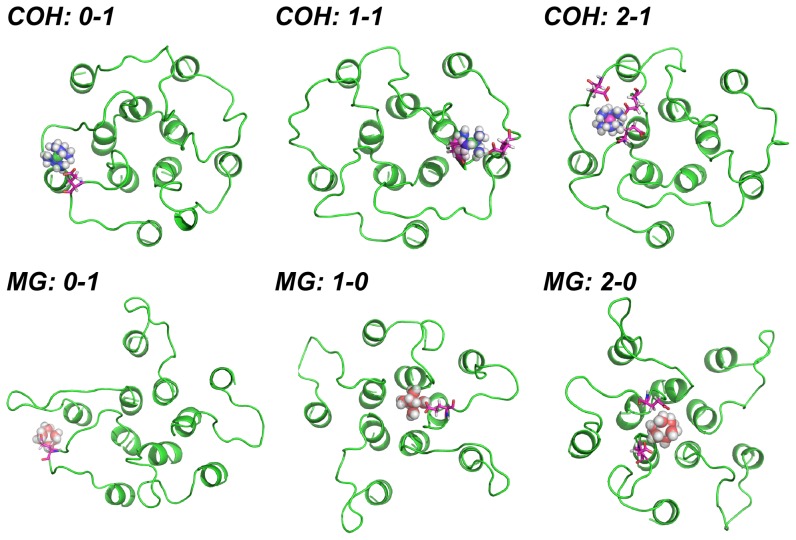
Snapshots taken from last 10 ns of ensemble trajectory at 315 K. The snapshots demonstrate the different patterns of interactions between cation and protein which are labelled in the top left corner of each snapshot. Cations and their interacting residues are highlighted. Upper panel shows the snapshots taken from COH system, and lower panel shows snapshots taken from MG system.

Moreover, the binding energy of ions and the loops were estimated with generalized-Born surface area (GBSA) model. The trajectory from 90 ns to 100 ns was split into 10 separated 10 ns-long trajectories where only 1 out of the 10 cations was present. The ten 10 ns-long trajectories were then concatenated into a new 100 ns trajectory, denoted as single-ion trajectory. Every frame of the new 100 ns single-ion trajectory was used for the GBSA analysis. The bound ions were then clustered according to the distances between them and E316 or E320. Each cluster is represented by two numbers, such as 1–2. The first number indicates the number of E316 residues in close contact with an ion, and the second number denotes the number of E320 residues which are close to the same ion. The clustering results were shown in Table 1. From the table, one can observe that the binding energy is mostly contributed by the electrostatic interactions. The numerical results also show that HexCo has a lower binding energy than Mg^2+^ when interacting with a same number of glutamic acid residues, which is in consistent with the previous PMF results. Further looking into the table, the 1-0 and 2-0 clusters have lower binding energy than 0–1 and 0–2 clusters in both COH and MG systems. This indicates that the E316 has a higher binding affinity than E320 for both HexCo and Mg^2+^ ions. The last column of Table 1 shows the population of each cluster. The first three most populated clusters for COH system is 0-1, 1-1 and 2-1, while for MG system is 1-0, 0-1 and 2-0. Snapshots to demonstrate the six different binding patterns were extracted and listed in Figure 9. Those structures were selected through two steps. First, the frames that belong to a particular cluster were extracted and their structures were fitted using protein heavy atoms. Second, a single linkage clustering for the pre-fitted structures was performed using the RMSD of the ion and the cluster centre was taken as the representative structure. E316 and E320 residues which were located within 0.5 nm from the ion are shown in sticks.

**Table 1:** Binding energy calculated based on implicit GBSA model.

*Cluster* a	*G_binding_* b	*E_GB_*	*E_vdw_*	*E_ele_*	*Population* c *(%)*
***COH system***					
0–1	−83.48±74.10	99.68	1.30	−184.46	35.5
0–2	−158.50±56.79	170.27	3.93	−332.70	3.3
1–0	−193.40±77.09	250.30	1.23	−444.93	7.8
1–1	−237.54±65.33	274.47	2.39	−514.40	16.7
1–2	−327.63±28.01	351.76	3.98	−683.37	0.4
2–0	−259.10±77.63	303.92	3.28	−566.30	9.6
2–1	−335.51±82.06	355.49	4.35	−695.35	13.4
***MG system***					
0–1	−33.44±29.78	40.86	0.67	−74.97	29.0
0–2	−68.15±49.95	165.29	−0.93	−232.51	0.5
1–0	−84.23±35.83	194.63	−3.11	−275.75	36.4
1–1	−127.13±36.39	263.33	−2.18	−388.28	11.4
1–2	−160.61±29.81	311.09	−4.30	−467.40	0.1
2–0	−108.07±38.07	236.18	−1.73	−342.52	15.2
2–1	−157.20±33.99	305.04	−0.71	−461.53	4.4

a Cluster *x – y* means the number of E316 residues which were located less than 0.5 nm from ions is x, and the number of E320 residues which were located less than 0.5 nm from ions is y.

b the unit for energy terms is kJ/mol.

c Population is the percentage of a cluster in the population where at least 1 E316 or E320 was located less than 0.5 nm from ions.

### Global binding pattern of ions

It is natural to ask how many ions can bind to the protein simultaneously since the protein is pentameric. To check it, the minimum distances from each Co atom of HexCo or Mg^2+^ atom to every residue of the protein were calculated from the ensemble trajectories at 315 K per frame. A residue was defined as an interacting-residue when the distance between the residue and the ion was less than 0.5 nm. And bound state was defined when there was at least one interacting-residue at the particular frame. The results are shown in Table 2 column 2. Surprisingly, there are almost 5 HexCo ions binding with protein in each frame, while only 3 for Mg^2+^ ions. The results indicate that ions can bind to the loops through several different binding sites.

**Table 2:** Bound states clustered based on distances between every ion to every residue.

*System*	*Average Number of Ions Bound*	*Populated Bound States*	*Residues involved in Binding* a b
***COH***	5.15±1.007	5 (42.1%)	E320(A), E320(B), E316(B)/E320(C), E316(C)/E320(E), E316(A)/E320(D)/E316(E)
		6 (26.4%)	E316(A), E320(B), E320(E), E320(A)/E320(D), E316(D)/E316(E), E316(A)/E316(D)/E320(D)/E316(E)
		4 (21.7%)	E316(A), E320(B), E320(A)/E320(D), E316(B)/E316(C)/E320(C)
***MG***	2.89±1.155	3 (39.3%)	E316(A), E316(C), E316(A)/F315(D)/E316(D)
		2 (27.2%)	E320(B), E316(D)
		4 (20.8%)	E320(B), E320(C), E320(E), E316(A)/E316(C)

a The letter inside brackets indicates the chain name for the residue.

b The comma “,” separates different bound states, the slash “/” separates different residues in a particular bound state.

The residues involved in the multiple binding sites were then investigated based on the distances calculated. First, the bound states were further clustered according to the number of ions bound per frame. Then the first 3 most populated bound states were extracted to find out the most common interacting-residues. The results are shown in Table 2 columns 3, and 4. Consistent with the results of average minimum distances from ion to residues, the most common interacting residues are E316 and E320 for both COH and MG systems. In COH system, other residues were barely seen to be interacting residues. In MG system, F315 was seen when number of bound ions was 3, suggesting its role in the binding as a cooperative binding site.

**Figure 10:**
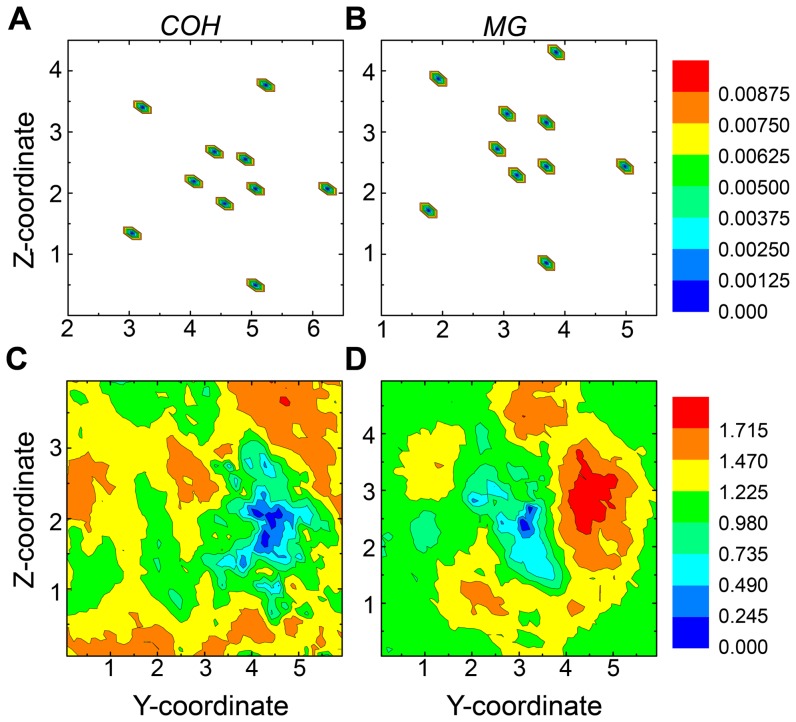
The 2-dimensional PMF on Y and Z Cartesian coordinates of different atoms. The free energy of the alpha carbon atoms of Y311 and P328 from COH system (A) and from MG system (B); The free energy of Cobalt atoms from COH system (C) and Mg atoms from MG system(D). The units of X-axis and Y axis are nm. The unit for free energy is kJ/mol.

To check where the multiple binding sites were located, we investigated the Cartesian coordinates of the ions. The coordinates of every frame were extracted from ensemble trajectory at 315 K. Since the system was built in the way that the molecular channel-axis was parallel to the X-axis of system, the Y and Z coordinates were chosen first to show the general distributions of ions. The Cartesian coordinates of the Cα atoms from Y311 and P328, 10 atoms in total, were also extracted. These atoms are constrained during the whole simulations and their coordinates were plotted here to directly show the position of the channel (Figures 10A and 10B). The 2-dimensional PMF methods were applied, and the free energy surface was shown in Figure 10. The standard errors for Figure 10A and 10B are both in the scale of 10^−5^ kJ/mol. And for Figure 10C and 10D, they are 0.03421 kJ/mol and 0.02101 kJ/mol respectively. From the Figure 10A, one observes the channel of COH system is mainly located in the region where y = 4.0–5.0 and z = 1.8–2.8. As to the HexCo ions, in Figure 10C, one finds the most highly sampled regions is located mainly in the channel region. As to the MG system, the channel region is around y = 3.0–4.0 and z = 2.4–3.4 (Figure 10B), where Mg^2+^ ions are most populated. From the above results, one can see that the HexCo and Mg^2+^ ions globally prefer binding to the loops located on the top of the inner circle formed by the TM1 domains, rather than those outside the outer circle formed by TM2, or in between the two domains. In comparison with the HexCo ions, Mg^2+^ ions sample much broader space on the Y-Z dimensions.

**Figure 11:**
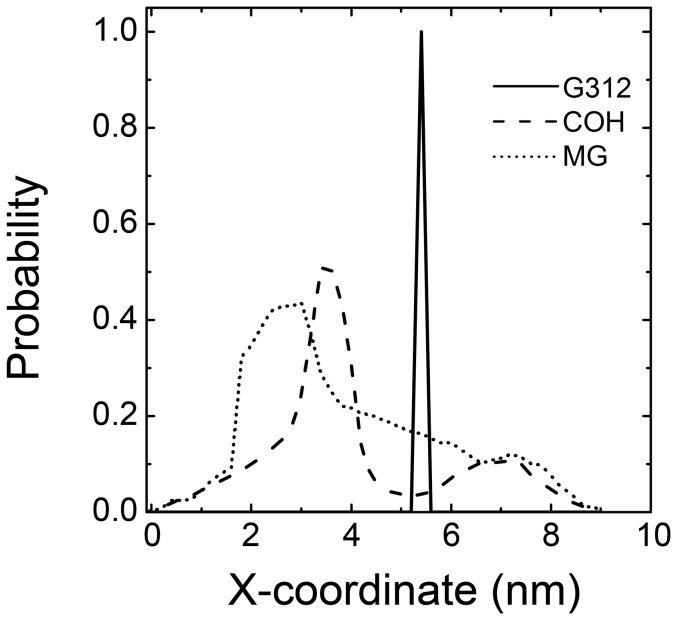
Distribution of X-coordinates of different atoms extracted from ensemble trajectory at 315 K. Black line represents the distribution of X-coordinates of alpha carbons of G312 from both systems. Blue line represents the distribution of X-coordinates of Co atoms from COH system. Red line represents the distribution of X-coordinates of Mg atoms from MG system.

In addition to the 2D-PMF on Y and Z dimensions, the X-coordinates were also investigated. The X-coordinates of the alpha carbon of G312 from both systems, Co atoms from the COH system and Mg^2+^ atoms from the MG system were extracted from the ensemble trajectory at 315 K. The distribution of these coordinates were calculated and plotted in Figure 11. The alpha carbon atoms of G312 residues were used to indicate the position of the channel mouth along the X-axis. The HexCo ions and Mg^2+^ ions show different distribution patterns along the X-axis as shown clearly in Figure 11. Most of HexCo ions were located closer to channel mouth than Mg ions. Conclusively, both HexCo and Mg^2+^ ions bind to loops on the top of the channel, and HexCo ions bind to loops closer to the channel mouth than Mg ions.

## Discussion and Summary

In this study, we explored the configuration of the flexible inter-helical loops of *T. maritima* CorA, and investigated the binding patterns of Mg^2+^ ions and HexCo ions to the loops which are characterized by the averaged minimum distances, the PMFs and the average number of bound ions.

Based on our study both E316 and E320 are the main players for Mg^2+^ and HexCo ions binding and on the whole loop there are multiple binding sites available. The side chains of E316 residues are pointing toward the central axis forming a “negatively charged nest” to ions, as suggested by Dalmas et al. (13). The E320 residues also form high amount of contacts with Mg^2+^ ions.

The inhibition mechanism of HexCo ion on the transportation of Mg^2+^ ions can be soundly explained. First of all, HexCo ions own a higher binding affinity than Mg^2+^ ions, which gives them a priority to bind the periplasmic loops over Mg^2+^ ions. Secondly, the number of HexCo ions binding on the loops simultaneously is as many as 5 in our model. The 5 ions could expel the Mg^2+^ ions away from the loops through electrostatic repulsion, resulting in incapable binding of Mg^2+^ ions. In addition, both ions prefer binding to the loops on the top of the channel with HexCo ions preferring binding closer to the mouth of the channel than Mg^2+^ ions. We realize that the absence of explicit lipid bilayer environment in our simulations neglects the lipid-water interface and the low-dielectric medium inside the lipid bilayer which may influence the ions distributions nearby. Fortunately the loop domains which are the focus of the current study are well above the lipid-water interface. The ions distribution within the loop domains may experience little perturbations from the lipid-water interface.

In a recent experiment, Moonmaw and Maguire mutated E281 and E285 on *Salmonella enterica* serovar Typhimurium CorA separately (relating to E316 and E320 of *T. maritima* CorA) and found the overall transportation efficiency was not affected too much [11]. Our simulation study predicted that mutated negatively charged residues will reduce the ions binding affinity. Thus the above experimental data provide a hint that the bottleneck of the overall transportation process may not come from the initial binding events but may resort to the downstream ions translocation through the channel.

Unfortunately, the simulation of the whole process of Mg^2+^ ions transportation through CorA is beyond the scope of the current study. We catch here the structural and energetic properties relating the initial binding of ions on the flexible loops. The one-dimensional free energy landscapes of ion binding (Figure 8C and 8D) are clearly funnel-like. Statistically ions move toward the mouth of the channel under a favourable environment which is constructed by the flexible loops and the aqueous medium.

A very recent work by Xia *et al.* [32] found that *T. maritima* CorA was not able to regulate the homeostasis of Mg ions. On the contrary, it could transport divalent cobalt ions with a high selectivity. Moreover they found that divalent cobalt ions could induce significant conformational changes of the protein during the transportation process, while Mg^2+^ cannot. Considering the same charge state and similar ionic radii (65 pm for Co^2+^ and 72 pm for Mg^2+^) [33], the initial binding patterns of Co^2+^ on the loops should be similar to that of Mg^2+^. However, the delicate energetic differences of hydration structures between Co^2+^ and Mg^2+^ ions may bring about the transportation selectivity of the CorA channel.

In summary we studied the initial binding of ions to the inter-helical loops of CorA. As to other possible functions of the loops, such as the dehydration of ions, transportation of ions from the binding sites into the channel and the induction of the open/close state transitions during transportation, further experimental and theoretical studies are needed.

## Supporting Information

Section S1 The radial distribution function of HexCo/Mg2+ ion - Oxygen in Water molecules.
(DOC)

Section S2 The PMF of the distances between HexCo/Mg2+ ion and the Glutamic acid side chain.
(DOC)

Figure S1 The RDF of Co atom - water Oxygen atoms (black) and Mg^2+^ atom– water Oxygen atoms (red).
(TIF)

Figure S2 The PMF of the distance between HexCo (black) or Mg^2+^ (red) and the glutamic acid side chain.
(TIF)

Table S1 The parameters for HexCo.
(DOC)

Table S2 Secondary structures composition calculated by DSSP at 315 K.
(DOC)

## References

[1] Maguire ME, Cowan JA (2002) Magnesium chemistry and biochemistry. Biometals 15: 203–210. 12206387 10.1023/a:1016058229972

[2] Belyaev ND, Budker VG, Gorokhova OE, Sokolov AV (1988) MG2+-DEPENDENT INTERACTION OF DNA WITH EUKARYOTIC CELLS. Molecular Biology 22: 1332–1337. 3252155

[3] Pittman YR, Valente L, Jeppesen MG, Andersen GR, Patel S, et al (2006) Mg2+ and a key lysine modulate exchange activity of eukaryotic translation elongation factor 1B alpha. Journal of Biological Chemistry 281: 19457–19468. 16675455 10.1074/jbc.M601076200

[4] Smith RL, Maguire ME (1998) Microbial magnesium transport: unusual transporters searching for identity. Molecular Microbiology 28: 217–226. 9622348 10.1046/j.1365-2958.1998.00810.x

[5] Silver S (1969) ACTIVE TRANSPORT OF MAGNESIUM IN ESCHERICHIA COLI. Proceedings of the National Academy of Sciences of the United States of America 62: 764–&. 4895213 10.1073/pnas.62.3.764 PMC223664

[6] Hmiel SP, Snavely MD, Miller CG, Maguire ME (1986) MAGNESIUM TRANSPORT IN SALMONELLA-TYPHIMURIUM - CHARACTERIZATION OF MAGNESIUM INFLUX AND CLONING OF A TRANSPORT GENE. Journal of Bacteriology 168: 1444–1450. 3536881 10.1128/jb.168.3.1444-1450.1986 PMC213658

[7] Lunin VV, Dobrovetsky E, Khutoreskaya G, Zhang RG, Joachimiak A, et al (2006) Crystal structure of the CorA Mg2+ transporter. Nature 440: 833–837. 16598263 10.1038/nature04642 PMC3836678

[8] Payandeh J, Pai EF (2006) A structural basis for Mg2+ homeostasis and the CorA translocation cycle. EMBO J 25: 3762–3773. 16902408 10.1038/sj.emboj.7601269 PMC1553185

[9] Eshaghi S, Niegowski D, Kohl A, Molina DM, Lesley SA, et al (2006) Crystal structure of a divalent metal ion transporter CorA at 2.9 angstrom resolution. Science 313: 354–357. 16857941 10.1126/science.1127121

[10] Niegowski D, Eshaghi S (2007) The CorA family: Structure and function revisited. Cellular and Molecular Life Sciences 64: 2564–2574. 17619822 10.1007/s00018-007-7174-z PMC11136245

[11] Moomaw AS, Maguire ME (2010) Cation Selectivity by the CorA Mg2+ Channel Requires a Fully Hydrated Cation. Biochemistry 49: 5998–6008. 20568735 10.1021/bi1005656 PMC2912426

[12] Hu J, Sharma M, Qin HJ, Gao FP, Cross TA (2009) Ligand Binding in the Conserved Interhelical Loop of CorA, a Magnesium Transporter from Mycobacterium tuberculosis. Journal of Biological Chemistry 284: 15619–15628. 19346249 10.1074/jbc.M901581200 PMC2708858

[13] Dalmas O, Cuello LG, Jogini V, Cortes DM, Roux B, et al (2010) Structural Dynamics of the Magnesium-Bound Conformation of CorA in a Lipid Bilayer. Structure 18: 868–878. 20637423 10.1016/j.str.2010.04.009 PMC2935691

[14] Fiser A, Sali A (2003) MODELLER: Generation and refinement of homology-based protein structure models. Macromolecular Crystallography, Pt D. San Diego: Academic Press Inc. pp. 461–+. 10.1016/S0076-6879(03)74020-8 14696385

[15] Van der Spoel D, Lindahl E, Hess B, Groenhof G, Mark AE, et al (2005) GROMACS: Fast, flexible, and free. Journal of Computational Chemistry 26: 1701–1718. 16211538 10.1002/jcc.20291

[16] Wang J, Cieplak P, Kollman PA (2000) How well does a restrained electrostatic potential (RESP) model perform in calculating conformational energies of organic and biological molecules? Journal of Computational Chemistry 21: 1049–1074.

[17] Hansmann UHE (1997) Parallel tempering algorithm for conformational studies of biological molecules. Chemical Physics Letters 281: 140–150.

[18] Hansmann UHE (1997) Parallel tempering algorithm for conformational studies of biological molecules. Chemical Physics Letters 281: 140–150.

[19] Hermans J, Berendsen HJC, Vangunsteren WF, Postma JPM (1984) A CONSISTENT EMPIRICAL POTENTIAL FOR WATER-PROTEIN INTERACTIONS. Biopolymers 23: 1513–1518.

[20] Shao Y, Molnar LF, Jung Y, Kussmann J, Ochsenfeld C, et al (2006) Advances in methods and algorithms in a modern quantum chemistry program package. Physical Chemistry Chemical Physics 8: 3172–3191. 16902710 10.1039/b517914a

[21] Frisch MJ, Trucks GW, Schlegel HB, Scuseria GE, Robb MA, et al. (2009) Gaussian 09, Revision B.01. Wallingford CT.

[22] Dupradeau FY, Pigache A, Zaffran T, Savineau C, Lelong R, et al (2010) The R.ED. tools: advances in RESP and ESP charge derivation and force field library building. Physical Chemistry Chemical Physics 12: 7821–7839. 20574571 10.1039/c0cp00111b PMC2918240

[23] Cheatham TE, Kollman PA (1997) Insight into the stabilization of A-DNA by specific ion association: spontaneous B-DNA to A-DNA transitions observed in molecular dynamics simulations of d[ACCCGCGGGT]2 in the presence of hexaamminecobalt(III). Structure (London, England: 1993) 5: 1297–1311. 10.1016/s0969-2126(97)00282-7 9351805

[24] MacKerell AD, Bashford D, Bellott M, Dunbrack RL, Evanseck JD, et al (1998) All-atom empirical potential for molecular modeling and dynamics studies of proteins. Journal of Physical Chemistry B 102: 3586–3616. 10.1021/jp973084f 24889800

[25] Hess B, Bekker H, Berendsen HJC, Fraaije J (1997) LINCS: A linear constraint solver for molecular simulations. Journal of Computational Chemistry 18: 1463–1472.

[26] Essmann U, Perera L, Berkowitz ML, Darden T, Lee H, et al (1995) A SMOOTH PARTICLE MESH EWALD METHOD. Journal of Chemical Physics 103: 8577–8593.

[27] Case DA, Darden TA, Cheatham, Simmerling CL, Wang J, et al. (2006) Amber 9.

[28] Onufriev A, Bashford D, Case DA (2004) Exploring protein native states and large-scale conformational changes with a modified generalized born model. Proteins-Structure Function and Bioinformatics 55: 383–394. 10.1002/prot.20033 15048829

[29] Kiriukhin MY, Collins KD (2002) Dynamic hydration numbers for biologically important ions. Biophysical Chemistry 99: 155–168. 12377366 10.1016/s0301-4622(02)00153-9

[30] Kucharski LM, Lubbe WJ, Maguire ME (2000) Cation hexaammines are selective and potent inhibitors of the CorA magnesium transport system. Journal of Biological Chemistry 275: 16767–16773. 10748031 10.1074/jbc.M001507200

[31] Yung-Chi C, Prusoff WH (1973) Relationship between the inhibition constant (KI) and the concentration of inhibitor which causes 50 per cent inhibition (I50) of an enzymatic reaction. Biochemical Pharmacology 22: 3099–3108. 4202581 10.1016/0006-2952(73)90196-2

[32] Xia Y, Lundback AK, Sahaf N, Nordlund G, Brzezinski P, et al (2011) Co(2+) Selectivity of Thermotoga maritima CorA and Its Inability to Regulate Mg(2+) Homeostasis Present a New Class of CorA Proteins. Journal of Biological Chemistry 286. 10.1074/jbc.M111.222166 PMC3091257 21454699

[33] Shannon R (1976) Revised effective ionic radii and systematic studies of interatomic distances in halides and chalcogenides. Acta Crystallographica Section A 32: 751–767.

