# Supplementary material for: Initial Binding of Ions to the Interhelical Loops of Divalent Ion Transporter CorA: Replica Exchange Molecular Dynamics Simulation Study
Source: PLoS One. 2012 Aug 30;7(8):e43872. doi: 10.1371/journal.pone.0043872 (PMC3431404; doi:10.1371/journal.pone.0043872)
Supplement: Section S1 — The radial distribution function of HexCo/Mg2+ ion - Oxygen in Water molecules. (DOC) [file pone.0043872.s001.doc]

### Section S1. The Radial Distribution Function of HexCo/Mg^2+^ ion - Oxygen in Water molecules.

The radial distribution function (RDF) of HexCo or Mg^2+^ ion with respect to water was used as a test of the force field parameters of the ions. The RDF was calculated according to following equation.

$$g_{ion-wat}(r)=\frac{\left\langle\rho_{wat}(r)\right\rangle}{\left\langle\rho_{wat}\right\rangle_{local}}=\frac{1}{\left\langle\rho_{wat}\right\rangle_{local}}\frac{1}{N_{ion}}\sum_{i\in ion}^{N_{ion}} \sum_{j\in wat}^{N_{wat}} \frac{\delta(r_{ij}-r)}{4\pi r^{2}}$$

Where, is the density of water at a distance *r* around the ion; $\left\langle\rho_{wat}\right\rangle_{local}$ is the density of water averaged over all spheres around the ion with radius *r_max_*, which is the half of the box length.

A HexCo or Mg^2+^ ion was placed in the centre of a cubic box with length of 3 nm, which was filled with SPC waters. 3 or 2 countering ions, CL-, were randomly placed into the box containing HexCo or Mg^2+^ ion respectively. The whole box was energy minimised for 10ps using steepest descent minimization, and equilibrated for 10ps under NPT ensemble with T=300K and P=1atm. The final production MD simulation was run for 10 ns under NPT ensemble with T=300K and P=1atm. The protocols used to perform the simulation here, including software used, methods used to treat long range interactions, *etc*, were the same as the protocols described in the section entitled “Models and Methods”.

The RDF of the Co atom in HexCo or Mg^2+^ atom with respect to the water oxygen atoms were plotted in Figure S1. The 1^st^ hydration shell of Mg^2+^ ion is located at the distance of 0.2 nm away from the ion, where the NH3 groups are located in HexCo ion. The 2^nd^ hydration shell of Mg^2+^ is located at the distance of 0.42 nm, which is slightly further than the 1^st^ hydration shell of HexCo ion, 0.39nm.
