# Supplementary material for: Initial Binding of Ions to the Interhelical Loops of Divalent Ion Transporter CorA: Replica Exchange Molecular Dynamics Simulation Study
Source: PLoS One. 2012 Aug 30;7(8):e43872. doi: 10.1371/journal.pone.0043872 (PMC3431404; doi:10.1371/journal.pone.0043872)
Supplement: Section S2 — The PMF of the distances between HexCo/Mg2+ ion and the Glutamic acid side chain. (DOC) [file pone.0043872.s002.doc]

### Section S2. The PMF of the distances between HexCo/ Mg^2+^ ion and the Glutamic acid side chain.

The PMF of the minimum distances between HexCo or Mg^2+^ ion and glutamic acid side chain was used to construct the free energy surface of ion binding to the glutamic acid side chain. The free energy was given according to equation 1 in section entitled “Models and Methods”.

A glutamic acid capped with ACE and NME, and a HexCo or Mg^2+^ ion which was located > 1 nm away from the glutamic acid surface initially were placed in a cubic box with length of 3 nm. The centre of mass of the residue-ion complex was treated as the centre of the cubic box. The boxes were solvated with SPC waters. Then 2 or 1 countering ions, CL-, were randomly placed into the box containing HexCo or Mg^2+^ ion respectively. The whole box was energy minimised for 10ps using steepest descent minimization, and equilibrated for 10ps under NPT ensemble with T=300K and P=1atm. The final production MD simulation was run for 30 ns under NPT ensemble with T=300K and P=1atm. The protocols used to perform the simulation here, including software used, methods used to treat long range interactions, *etc*, were the same as the protocols described in the section entitled “Models and Methods”.

The free energy surface constructed using PMF was shown in Figure S2. The free energies were normalized at the distance of 1.5nm, which is half of the box length. For the HexCo ion system, the global minimum is located at 0.37 nm, and the free energy value was estimated to be -9.5 kJ/mol. For the Mg^2+^ ion system, the global minimum is located at 0.39 nm, and the free energy value was estimated to be -3.2 kJ/mol. The binding of HexCo ion to glutamic acid is stronger than that of Mg^2+^ ion, with the free energy -6.3 kJ/mol lower.
