# Supplementary figures and images for: Initial Binding of Ions to the Interhelical Loops of Divalent Ion Transporter CorA: Replica Exchange Molecular Dynamics Simulation Study
Source: PLoS One. 2012 Aug 30;7(8):e43872. doi: 10.1371/journal.pone.0043872 (PMC3431404; doi:10.1371/journal.pone.0043872)

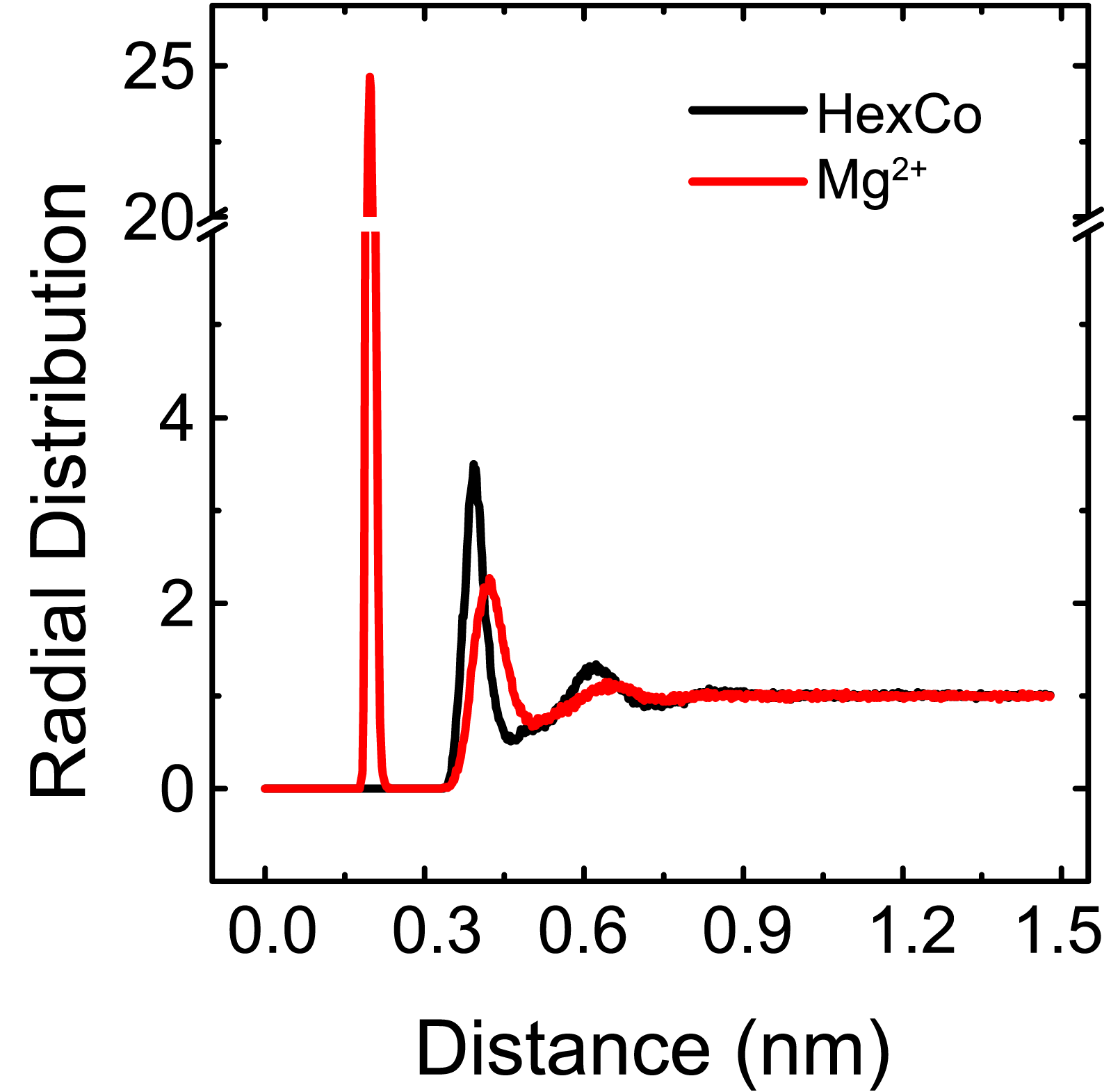

Supplement: Figure S1 — The RDF of Co atom - water Oxygen atoms (black) and Mg2+ atom– water Oxygen atoms (red). (TIF) [file pone.0043872.s003.tif]

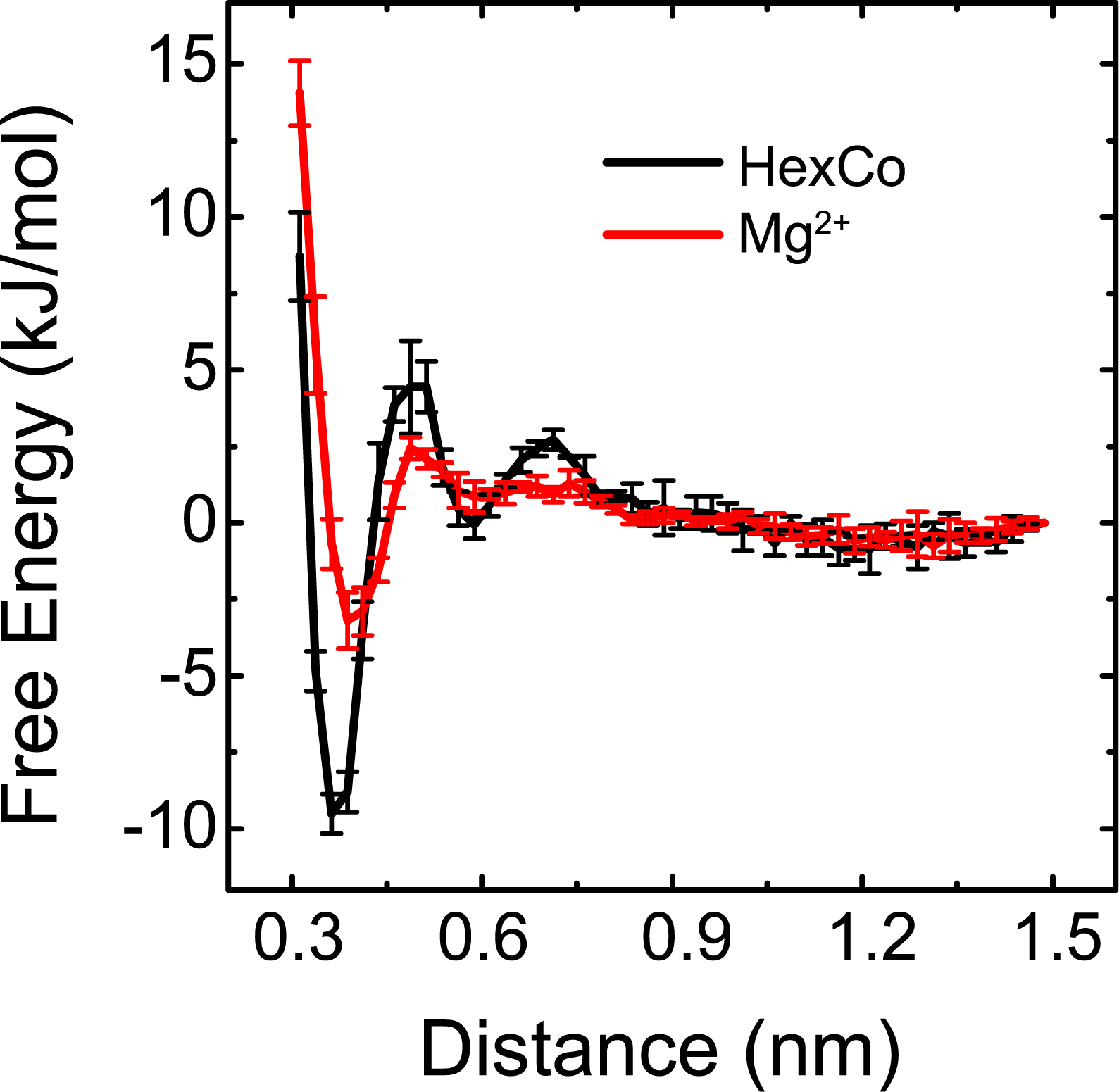

Supplement: Figure S2 — The PMF of the distance between HexCo (black) or Mg2+ (red) and the glutamic acid side chain. (TIF) [file pone.0043872.s004.tif]
