# Supplementary material for: Initial Binding of Ions to the Interhelical Loops of Divalent Ion Transporter CorA: Replica Exchange Molecular Dynamics Simulation Study
Source: PLoS One. 2012 Aug 30;7(8):e43872. doi: 10.1371/journal.pone.0043872 (PMC3431404; doi:10.1371/journal.pone.0043872)
Supplement: Table S1 — The parameters for HexCo. (DOC) [file pone.0043872.s005.doc]

**Table S1**: The parameters for HexCo.

| ***Atoms*** | ***Charges*** | ***Sigma (Å)*** | ***Epsilon (kJ/mol)*** |
| --- | --- | --- | --- |
| Co | 1.062 | 1.41225 | 3.7434200 |
| N | -0.784 | 3.25000 | 0.7112800 |
| H | 0.369 | 1.06908 | 0.0656888 |

| ***Bonds*** | ***r_eq_ (Å)*** | ***K_bond_ (kJ/mol/nm^2^)*** |
| --- | --- | --- |
| Co-N | 2.070 | 25104 |
| N-H | 1.037 | 41840 |

| ***Angles*** | ***θ_eq_ (degree)*** | ***K_angle_ (kJ/mol/rad^2^)*** |
| --- | --- | --- |
| N-Co-N | 90 | 585.76 |
| N-Co-N | 180 | 585.76 |
| Co-N-H | 109.5 | 292.88 |
