# Supplementary material for: Initial Binding of Ions to the Interhelical Loops of Divalent Ion Transporter CorA: Replica Exchange Molecular Dynamics Simulation Study
Source: PLoS One. 2012 Aug 30;7(8):e43872. doi: 10.1371/journal.pone.0043872 (PMC3431404; doi:10.1371/journal.pone.0043872)
Supplement: Table S2 — Secondary structures composition calculated by DSSP at 315 K. (DOC) [file pone.0043872.s006.doc]

**Table S2**. Secondary structures composition calculated by DSSP at 315K.

| ***SYSTEM*** | ***Coil*** | ***Turn*** | ***Beta*** | ***Helix*** |
| --- | --- | --- | --- | --- |
| ***LOOP*** | 71.3 (0.18) | 18.5 (0.13) | 1.7 (0.045) | 8.5 (0.11) |
| ***COH*** | 72.9 (0.22) | 16.5 (0.16) | 0.8 (0.060) | 9.8 (0.13) |
| ***MG*** | 71.9 (0.25) | 19.1 (0.19) | 1.3 (0.060) | 7.7 (0.19) |
